# Lipophilic but not hydrophilic statin therapy improves cardiodynamics without affecting skeletal muscle morphology or function in geriatric Wistar rats

**DOI:** 10.1371/journal.pone.0339194

**Published:** 2026-01-02

**Authors:** Irena A. Rebalka, Razan Alshamali, Tiffany L. vanLieshout, Molly A. Gingrich, Matthew R. Marlatt, Vladimir Ljubicic, Jeremy A. Simpson, Thomas J. Hawke

**Affiliations:** 1 Department of Pathology and Molecular Medicine, McMaster University, Hamilton, Ontario, Canada; 2 Department of Human Health and Nutritional Sciences, University of Guelph, Guelph, Ontario, Canada; 3 Department of Kinesiology, McMaster University, Hamilton, Ontario, Canada; China Medical University, TAIWAN

## Abstract

While older adults are the most prominent human cohort currently necessitating or receiving cholesterol-lowering statin therapy, most preclinical testing of the efficacy and side effects that accompany statin use are conducted on juvenile adult rodents. Furthermore, varying pharmacodynamics between hydrophilic and lipophilic statins may lead to different therapeutic outcomes. The purpose of this study was to compare the efficacy, specifically modifications to cardiovascular parameters, and safety, namely presence or absence of a pathological skeletal muscle phenotype, of hydrophilic rosuvastatin (RSV) versus lipophilic atorvastatin (ATV) therapy in a geriatric rodent model. Briefly, 25- to 27-month-old Wistar rats were provided control, RSV, or ATV treatment for 30 days. Cardiac hemodynamics, muscle functional testing, open-field behaviours, and skeletal muscle morphology were evaluated. Whole-heart weight was lower in ATV-treated than in RSV-treated animals. Heart rate, rate of left ventricle pressure change, and arterial pressure were lowered by ATV but not RSV. These results are hypothesized to suggest attenuation of age-related sympathetic overactivity with ATV treatment. No differences in muscle histology, function, or open-field testing behaviours were observed between groups, indicating the absence of an overt statin-associated skeletal muscle phenotype. Overall, in geriatric rodents that more appropriately mimic the age of humans that are prescribed statin therapy, lipophilic ATV, but not hydrophilic RSV, had positive effects on cardiac function without negatively impacting the skeletal muscle.

## Introduction

The class of HMG-CoA (3-hydroxy-3-methylglutaryl-CoA) reductase inhibitors, colloquially known as statins, are front-line therapy for managing hypercholesterolemia and reducing atherosclerotic cardiovascular disease (ASCVD) risk by inhibiting cholesterol production. Depending upon the dose and type of statin prescribed, up to a 46% reduction in total cholesterol and a 60% reduction in low-density lipoprotein cholesterol have been reported, greatly improving cardiovascular outcomes [1–4].

While all statins share a common mechanism of action, they differ in terms of their solubility; being on a spectrum from hydrophilic (e.g., rosuvastatin; RSV) to lipophilic (e.g., atorvastatin; ATV). Research indicates that lipophilic statins are associated with better outcomes in the prevention of cardiovascular events, including lower rates of mortality and hospitalization, when compared to hydrophilic statins [5,6]. Lipophilic statins are, however, also more closely associated with peripheral side-effects due to their ease of entry into extrahepatic tissues. In contrast, hydrophilic statins primarily target the liver and do not readily enter peripheral tissues [7]. As such, studies that directly compare lipophilic and hydrophilic statin therapy in both their therapeutic benefits and peripheral side effects are required.

The peripheral impact of statin prescription on skeletal muscle is notable, particularly for older adults who are more susceptible to the well-described muscle-related side effects of statins. [7,8]. Statin-associated muscle symptoms (SAMS), including muscle pain, weakness, and cramping, are the most frequently reported complaint, accounting for upwards of 72% of all reported statin side-effects [9]. This is of increasing concern, as the prescription of statins continues to rise worldwide, especially in the aging population with distinct therapeutic needs [10–12].

Despite the prominence of statin prescription in older adults, as well as the susceptibility of this population to SAMS and other comorbid conditions, preclinical testing of the side effects that accompany statin usage most commonly utilize juvenile or young adult rodents [13]. With respect to preclinical studies investigating statin prescription, a systematic review reports the mean age of mice used to be 12.5 weeks old; the equivalent of 20 human years [14,15]. Similarly, the mean age of rats was reported as nine weeks, equivalent to approximately five human years [15,16]. As the equivalent geriatric human age is not reached until approximately 24 months of rodent life [14,16], it is evident that these preclinical study populations are not adequately representing the most common clinical population of statin users or their associated physiological obstacles. The urgency for the use of appropriate preclinical models is evident, and is clearly supported by the release of a scientific statement by the American Heart Association to provide guidance to researchers for the appropriate development and execution of animal atherosclerosis studies [17].

The purpose of this study was to compare the effects of hydrophilic RSV and lipophilic ATV on the cardiodynamics of an aged rodent model, reflecting the cohort of individuals most widely requiring and receiving statin therapy. Additionally, we extended this investigation into the skeletal muscle, the tissue with the most reported peripheral adverse events. This two-pronged approach allowed for the simultaneous investigation of statin efficacy (in its intended organ system) and safety (pertaining to SAMS). This study provides a direct comparison between two statins with varying lipophilicity, which may help guide future clinical narratives pertaining to optimal statin prescription within the aging population, utilizing a more appropriate preclinical model for the evaluation of statin therapy.

## Materials and methods

### Animal handling and treatment

Male Wistar rats were aged in-house until 25–27 months of age. Animals were housed in a temperature-regulated room on a 12:12 hour light-dark cycle with food and water available *ad libitum*. Experimentation was approved by the University of Guelph Animal Care Committee in accordance with the Canadian Council for Animal Care guidelines. All geriatric rats were randomly assigned to receive oral control (CON; 0.5 tsp Nutella (Ferrero SpA; Alba, Italy)), rosuvastatin (RSV; 50 mg/kg; Sivem Pharmaceuticals; Saint-Laurent, QC, Canada; in CON), or atorvastatin (ATV; 50 mg/kg; Sivem Pharmaceuticals; in CON) treatment each morning for 30 consecutive days. Each animal consumed the full treatment dose each day. Baseline testing was conducted prior to treatment initiation, and post-treatment testing was conducted immediately following the 30-day treatment period. A 30-day treatment period was selected as literature has previously shown detectable biochemical changes (days to two weeks), histopathological changes (as early as ten days), and functional deficits (two to three weeks) within this range of time following initiation of statin administration in rodents [18–21].

### Cardiac testing

Briefly, rats were anesthetized with a combination of 2% isoflurane in oxygen and kept at 37˚C with a water-filled heating blanket. Hemodynamic function was evaluated using a 1.2F catheter (FTS-1211B-0018; Scisense Inc.; London, ON, Canada) advanced through the right common carotid artery into the left ventricle (LV). LV pressure readings were digitized at 2 kHz and recorded using iWorx analytic software (LabScribe2; Dover, NH, USA) for off-line analysis. Heart rate (HR), maximal LV systolic pressure, minimal LV pressure, LV end-diastolic pressure (EDP), maximal and minimal rates of change in pressure (dP/dt Max and dP/dt Min, respectively) were computed using in-house software [22,23]. After hemodynamic evaluation, rats were euthanized by exsanguination, and tissues were harvested and stored for analysis.

### Muscle functional testing

Forelimb grip strength was measured as previously detailed [24,25]. Briefly, all animals were acclimatized to the protocol 24-hours prior to testing. During data collection, each rat performed three successive grip strength attempts and was subsequently returned to their cage for a one-minute rest period. This procedure was repeated five times for a total of 15 grip strength attempts per animal. The average of the three highest successive grip strength values were normalized to body weight (grams) to determine maximum grip strength. Fatigue was determined by calculating the decrement between the average of the first two series of attempts (1 + 2 + 3 = A, 4 + 5 + 6 = B) and the last two series of attempts (10 + 11 + 12 = C, 13 + 14 + 15 = D) using the formula: (C + D)/(A + B).

### Open-field testing

Open-field activity was measured using an open-field Opto-Varimex-5 Auto-Track (Columbus Instruments; Columbus, OH, USA) as previously described [26,27]. All animals were acclimatized to the position tracking chamber at least 24 hours prior to the five-minute data collection session. A variety of activity measures were recorded, including distance travelled, speed, ambulatory time, stereotypic time, rearing events, and time spent in the center or edge of the chamber.

### Evaluation of muscle morphology

Histological cross-sections of the tibialis anterior muscle were stained via standard protocols for hematoxylin and eosin (minimum feret diameter, minimum 150 adjacent muscle fibers per animal; central nucleation, entire muscle cross-section evaluated), as well as picrosirius red (collagen content, entire muscle cross-section evaluated). All images were captured using a Nikon Eclipse 90i microscope (Nikon Inc.; Melville, NY, USA) and analysed using NIS-Elements AR software (v4.6; Nikon Inc.).

### Statistics

All statistical tests were conducted using GraphPad Prism Version 10.2.3 (GraphPad Software; San Diego, CA, USA). For all analyses shown below, Brown-Forsythe test for normalcy was conducted and passed. A significant outlier, if any, was removed using Grubbs’ test. Statistical tests conducted for each method of analysis are detailed in the respective figure legend. When an ANOVA was conducted, Tukey’s multiple comparison test was employed as the post-hoc evaluation. Pearson Correlation Coefficients are reported with two-tailed p values. Statistical significance is denoted where appropriate at p < 0.05. All data presented within the text as well as graphs showing individual data points with encapsulating bars depict the group mean ± SEM.

## Results

### Pre- and post-study body mass did not differ between CON, RSV, or ATV-treated animals. Cardiac weight was lower in ATV-treated than in RSV-treated animals

**Fig 1:**
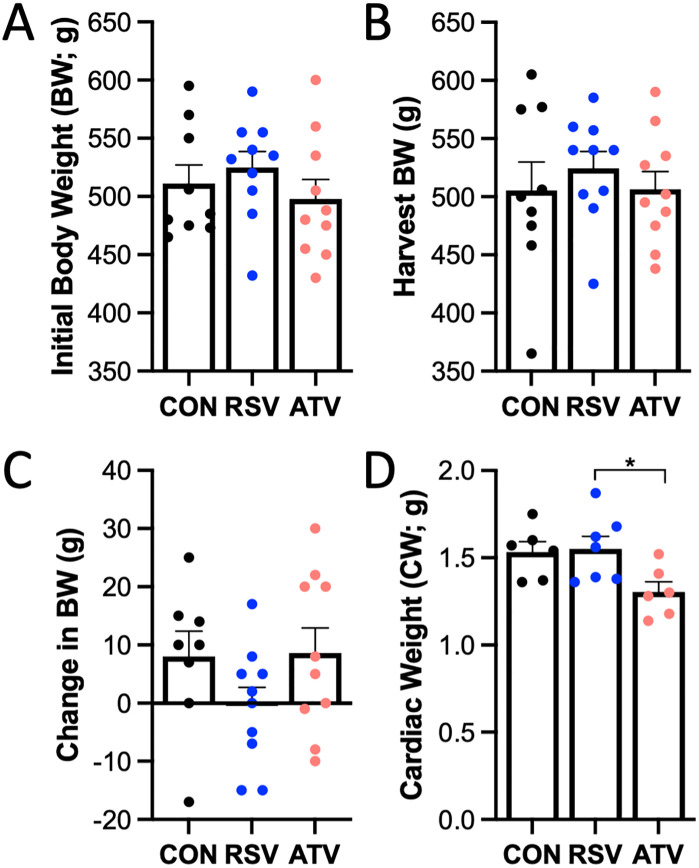
Body mass of geriatric rats was not affected by 30 days of statin treatment. **Cardiac weight was lower in rats treated with ATV than in those treated with RSV.** No differences in **(A)** body weight prior to study initiation, **(B)** harvest body weight following study completion, or **(C)** change in body weight during the study timeline were observed between groups. **(D)** Cardiac weight was significantly different between RSV and ATV treatment groups following 30 days of treatment. * = p < 0.05 via one-way ANOVA. All data are represented as mean ± SEM.

Prior to treatment administration, no differences in body weights were observed between treatment groups (Fig 1A; p = 0.47). After 30 days of CON, RSV, or ATV treatment, both harvest (study completion) body weight (Fig 1B; p = 0.71) and change in body weight from study initiation (Fig 1C; p = 0.20) remained consistent between groups. Absolute cardiac weight at study completion was significantly lower in ATV than RSV-treated animals, and no significant between-group differences were observed otherwise (Fig 1D).

### ATV but not RSV treatment lowered heart rate and rate of LV pressure change

**Fig 2:**
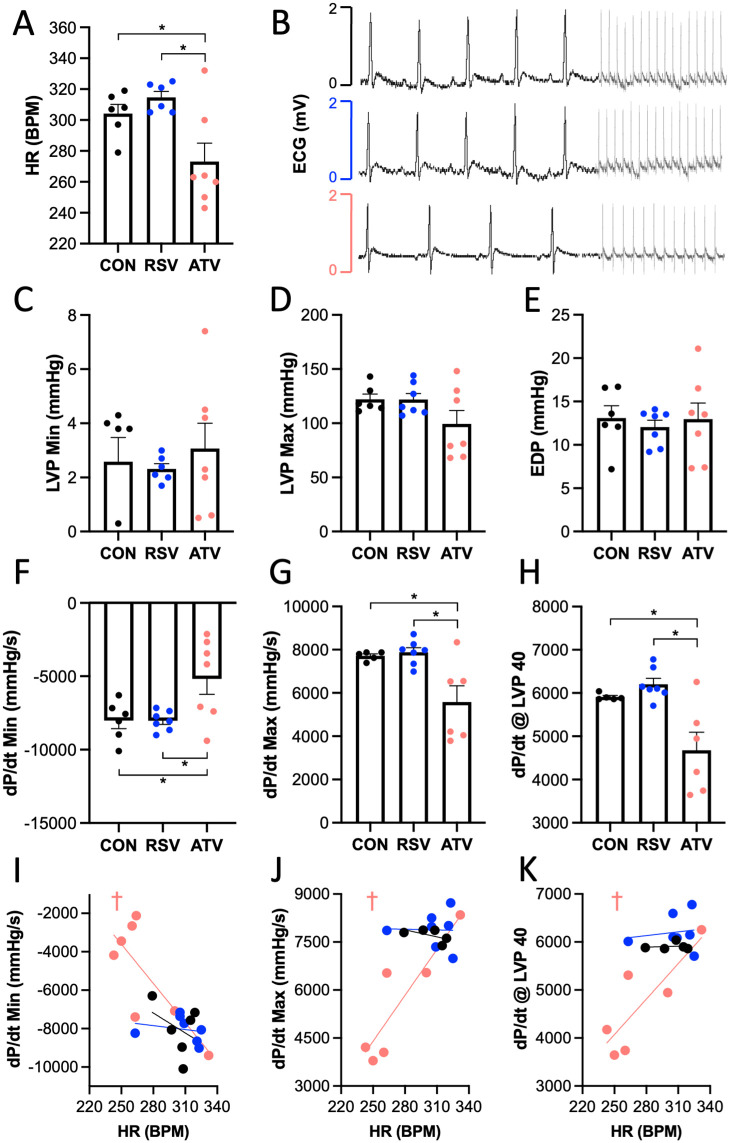
ATV, but not RSV, improved cardiodynamics. Following 30 days of treatment, **(A)** resting HR (BPM; beats per minute) was significantly decreased within the ATV treatment group when compared to both CON and RSV treatment groups. **(B)** This is depicted in representative electrocardiogram (ECG; mV; millivolts) tracings. **(C-E)** No difference in minimum cardiac pressure (LVP Min), maximum left ventricular pressure (LVP Max), or EDP was observed between groups. Within the LV, **(F)** the rate of relaxation as well as **(G)** the rate of contraction was significantly decreased within the ATV treatment group when compared to both CON and RSV treatment groups. **(H)** This trend for the rates of pressure change within the LV persisted independent of afterload (prior to aortic valve opening) at LVP 40 (40 mmHg pressure within the LV). **(I-K)** Strong, significant correlations between HR and rates of relaxation, contraction, and contraction prior to aortic valve opening were observed within the LV, but only with ATV treatment. * = p < 0.05 via one-way ANOVA. † = p < 0.05 via Pearson’s Correlation Coefficient, matching the colour of the group in which the significant correlation is present. For graphs A and C-H, all data are represented as mean ± SEM. For graphs I-K, simple linear regression for each treatment group is plotted along with all individual data points.

Thirty days of ATV treatment significantly reduced HR in geriatric rats by 10% and 13%, respectively, relative to CON and RSV treatment (Fig 2A,B). No difference in LVP Min was observed between groups (p = 0.78; Fig 2C). No difference in maximum left ventricular pressure (LVP Max) was observed between groups (p = 0.13; Fig 2D). No difference in EDP was observed between groups (p = 0.86; Fig 2E). There was, however, a significant reduction in the rate of change of pressure in the left ventricle (dP/dt Min, dP/dt Max, and dP/dt @ LVP40) in ATV-treated animals relative to both RSV and CON treatment, with no differences observed between CON and RSV treatment (Fig 2F-H). dP/dt Min and dP/dt Max and are indices of relaxation and contraction, respectively, and are dependent on HR, preload and afterload. dP/dt @ LVP40 indicates the rate of change of pressure in the left ventricle before aortic valve opening. There was a strong negative correlation between dP/dt Min and HR in only ATV-treated animals (CON r(18)= −0.37, p = 0.47; RSV r(18)= −0.22, p = 0.64; ATV r(18)= −0.77, p = 0.04; Fig 2I), indicating that a more rapid heart rate was associated with a more rapid rate of relaxation. There was a strong positive correlation between dP/dt Max and HR in only ATV-treated animals (CON r(18)= −0.57, p = 0.31; RSV r(18)= −0.03, p = 0.95; ATV r(18)= 0.89, p = 0.02; Fig 2J), indicating that a more rapid heart rate was associated with a more rapid rate of contraction. There was also strong positive correlation between dP/dt @ LVP 40 and HR in only ATV-treated animals (CON r(18)= 0.15, p = 0.81; RSV r(18)= 0.15, p = 0.75; ATV r(18)= 0.83, p = 0.04; Fig 2K), indicating that a more rapid heart rate was associated with a more rapid rate of contraction prior to the opening of the aortic valve and the initiation of ventricular systole.

### Arterial pressure was significantly reduced by ATV but not RSV treatment

**Fig 3:**
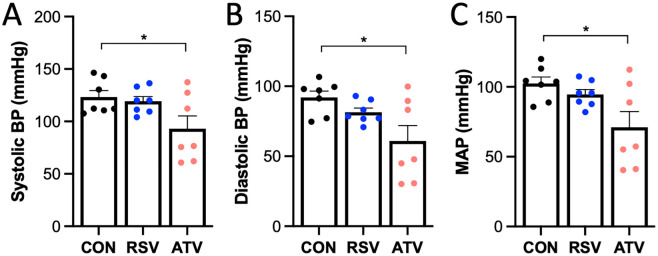
Arterial pressure was lowered by ATV but not RSV treatment. When compared to CON counterparts, **(A)** systolic BP, **(B)** diastolic BP, and **(D)** MAP were significantly attenuated by 30 days of ATV, but not RSV treatment. * = p < 0.05 via one-way ANOVA. All data are represented as mean ± SEM.

When compared to CON, a significant 24% decrease in systolic and 34% decrease in diastolic blood pressures (BP) were observed only with 30-days of ATV treatment (Fig 3A,B). Similarly, when compared to CON, mean arterial pressure (MAP) was significantly attenuated by 31% with ATV, but not RSV treatment (Fig 3C).

### Skeletal muscle morphology was not affected by RSV or ATV treatment

**Fig 4:**
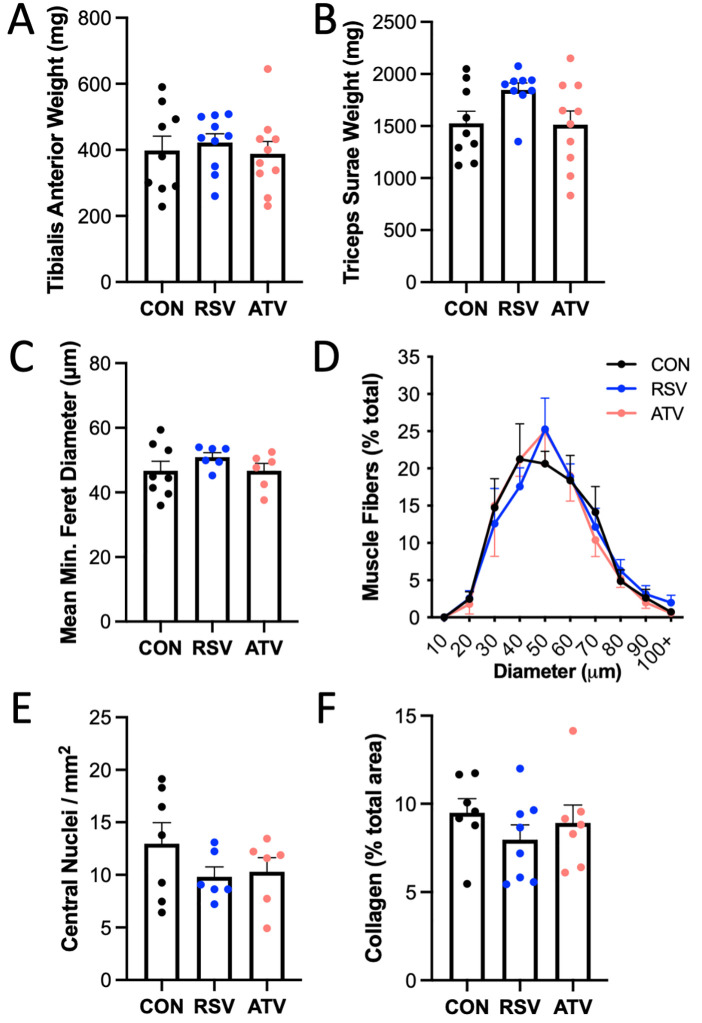
Overall skeletal muscle morphology was not affected by statin usage. No significant difference in **(A)** tibialis anterior muscle or **(B)** triceps surae muscle group (gastrocnemius, plantaris, soleus) weight was observed between treatment groups at harvest. **(C)** Mean minimum feret diameter and **(D)** histogram of mean minimum feret diameter confirm no difference in muscle fiber size between groups. When comparing all groups, no changes in the amount of **(E)** regenerating fibers, represented by the presence of central nucleation, or **(F)** percentage total collagen area were observed in skeletal muscle cross-sections. No significant differences, as tested via ANOVA; p < 0.05. All data are represented as mean ± SEM.

No significant differences in tibialis anterior or triceps surae muscle group (consisting of gastrocnemius, plantaris, and soleus muscles) weights were observed between treatment groups at the time of harvest (Fig 4A,B; p = 0.79 and p = 0.08, respectively). Mean muscle fiber minimum feret diameter remained similar between animals treated with CON, RSV, or ATV (Fig 4C; p = 0.41). Similarly, when clustered by size, the proportion of larger or smaller muscle fibers between treatment groups was comparable (Fig 4D; p = 0.37). No difference in central nucleation (indicating no difference in the degree of current muscle damage or repair) per area (Fig 4E; p = 0.31) or variability in the presence of collagen within the whole sectioned muscle (indicating difference in the degree of fibrosis; Fig 4F; p = 0.47) was observed. This collectively suggests no pathological indices within the skeletal muscle with RSV or ATV treatment.

### RSV and ATV treatment did not affect open-field testing behaviours or muscle performance

**Fig 5:**
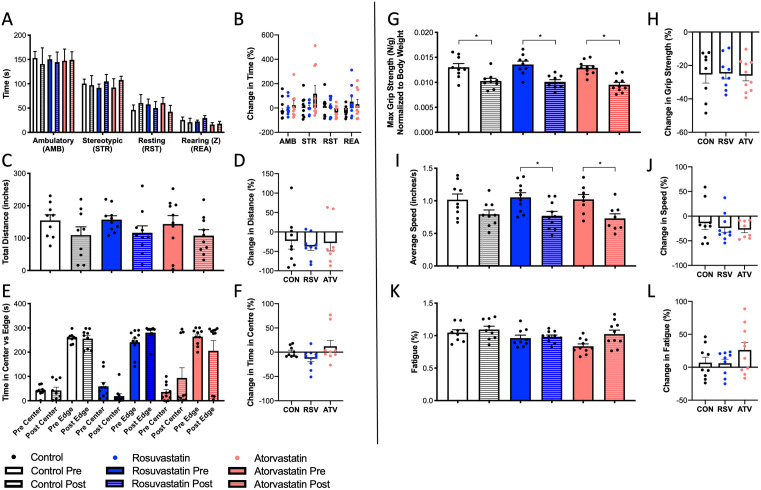
Open-field behaviours and muscle functional testing were not affected by statin usage. **(A)** No observable difference in activity was observed between treatment groups before or after statin therapy. **(B)** When each animal’s post-treatment activity was normalized to their initial behaviour, no differences between treatment groups persisted for all activity types. **(C)** No observable difference in total distance traveled during open-field testing was observed between treatment groups before or after statin therapy. **(D)** When each animal’s post-treatment distance traveled was normalized to their pre-treatment distance traveled during open field testing, no differences between treatment groups were observed. **(E)** Animals in each group spent comparable times in the center and edge of the field-testing area both before and after treatment. **(F)** When time in the center of the field-testing area was normalized to pre-treatment time for each individual animal, no differences between treatment groups were observed. **(G)** All treatment groups exhibited a significant decrease in maximum grip strength (N/g normalized to body weight) following 30-days of treatment, however, (H) there was no between-group difference in the magnitude of this decline. **(I)** Following study completion, RSV and ATV-treated animals exhibited a significant decrease in average travel speed during observation, however, **(J)** when comparing all treatment groups, there was no difference in the magnitude of this decline. **(K)** There were no between-group differences in pre-to-post treatment fatigue or **(L)** change in fatigue magnitude between groups. * = p < 0.05 via one-way ANOVA at each treatment time and test metric and paired two-tailed t-test within pre- and post-treatment metrics within each group. All data are represented as mean ± SEM.

During open-field testing, no significant differences in activity (ambulatory, stereotypic, resting, rearing), total distance traveled, or time in center versus edge were observed prior to or following any treatment (Fig 5A,C,E, respectively). When each animal’s post-treatment metrics were normalized to their pre-treatment metrics, again, there were no effects on activity, distance traveled, or time in center versus edge (Fig 5B,D,F, respectively). While maximum grip strength was significantly attenuated following CON (−23%), RSV (−29%), and ATV (−23%) treatment (Fig 5G), the magnitude of this decline was not affected by treatment type (Fig 5H; p = 0.97). Similarly, while average speed decreased by 23% in CON, and was significantly reduced by RSV (−27%) and ATV (−28%) treatment (Fig 5I), the magnitude of this decline did not differ between groups (Fig 5J; p = 0.52). Despite declines in grip strength and average locomotor speed, no between-group differences in pre-to-post treatment fatigue (Fig 5K) or change in fatigue following treatment (Fig 5L; p = 0.12) were observed.

## Discussion

Though statin prescription has become the standard of care for millions of aging individuals worldwide [10–12], preclinical testing of the efficacy and mechanisms underlying statin therapy is conducted in young, healthy rodents [15]. This introduces a facet of uncertainty when extrapolating preclinical inferences of statin prescription on cardiovascular benefits and systemic challenges to an aging human population. In the present study, we addressed this limitation by studying the impact of hydrophilic versus lipophilic statin administration on cardiodynamic and skeletal muscle metrics in geriatric rats, evaluating both therapeutic efficacy and peripheral complications. Our findings indicate that lipophilic ATV, but not hydrophilic RSV administration, improved metrics of cardiac function and reduced BP when compared to geriatric animals receiving no statin therapy. Furthermore, no adverse modifications to overt skeletal muscle health, function, or overall animal behaviour were observed with any statin administration, indicating an absence of peripheral complications that would prohibit therapeutic administration.

An important aspect of this study was the utilization of a rodent model that reflected attributes of the clinical population most likely on statin therapy [10–12]. Accordingly, our geriatric rat model had multiple phenotypic aspects that differentiated it from the younger rodent models often used in statin research studies. First, a significant loss of forelimb grip strength was observed over the 30-day experimental period, a phenomenon that is not observed in younger rats [28,29]. Additionally, the collective maximum grip strength of all animals within this study at baseline was 0.013 N/g, which declined to 0.0099 N/g at study completion. This is notably lower than previously reported grip strength in younger male Wistar rats; approximately 0.057 N/g at 14 weeks old, and 0.021 N/g at 17 months old [28,30]. Second, total serum cholesterol evaluated in the current model at the time of harvest (CON 121.5 ± 12.77, RSV 138.7 ± 6.89, ATV 144.7 ± 8.66 mg/dl) was similar in magnitude to levels reported in young cholesterol-fed hypercholesterolemic rats and dyslipidemic humans [31–33]. This is hypothesized to have occurred due to reduced cholesterol breakdown and a decrease in hepatic cholesterol clearance [34]. A limitation of this study, which is consistently observed in preclinical research involving rats on a standard chow diet, is the negligible reduction in serum cholesterol with statin therapy. This indicates that a mechanism other than a reduction in circulating cholesterol is responsible for the cardioprotection observed herein [15,35,36].

Focusing on cardiodynamic modifications, the superiority observed herein with lipophilic over hydrophilic statin therapy has previously been reported in patients with established heart failure [6,37,38]. Of particular importance within our results were the observed ATV-mediated reductions in HR, rates of LV contraction and relaxation, and the restoration of the relationship between these factors – independent cardiovascular components that are negatively impacted by sympathetic hyperactivity. Though a reduction in the rate of pressure could be indicative of impaired contractility, altered compliance, or negative changes in preload and afterload, this could also be viewed as a positive adaptation in the geriatric heart, where sympathetic nerve overactivity is widely implicated in the increased risk of ASCVD [39–41]. The speculated presence of, and decrease in sympathetic overactivity by ATV treatment may also be supported by our observed attenuation of cardiac mass with ATV treatment; indicating a delay in hypertrophic cardiac remodeling [42].

Consistent with the restored cardiodynamics observed with ATV treatment, significant reductions in HR, BP, and MAP were also only observed with ATV treatment in the current investigation. Previous work also details the diastolic BP-lowering properties of ATV when compared to other statins, including RSV, in hypertensive and dyslipidemic adults with a mean age of approximately 65 years [43]. In a new meta-analysis detailing the effects of statin therapy on BP in humans, ATV, but not RSV, was associated with decreased systolic BP, although correlations to cardiovascular health were not reported [44]. Forming a connection with the cardiodynamic modifications discussed above, lipophilic statins (including ATV) have been shown to reduce post-ganglionic efferent muscle sympathetic nerve activity by up to 29% in patients with hypertension and heart failure [45]. In the heart, this attenuation can lead to a decrease in heart rate, and in the vasculature, it can lead to reduced vasoconstriction, and reprieve from hypertension. While we acknowledge that there is an absence of sympathetic activity quantification in the current study, this limitation and hypothesis of mechanism of action certainly provides a foundation for future investigations.

Our work, as well as the aforementioned support, suggests functional cardiodynamic maintenance or restoration by ATV in the presence of cardiac dysfunction. Literature noting an absence of change in cardiac parameters with statin treatment in healthy rats can be contrasted with improvements in stroke volume, ejection fraction, cardiac output, BP, and vascular fibrosis following statin treatment in unhealthy rats [36]. Furthermore, additional studies reveal statistically insignificant reductions in BP in younger Wistar rats fed standard chow diet, even with ATV treatment [15,46]. Simply put, we believe that therapeutic functionality is observed when aberrant function is first evident; further supporting the use of a geriatric preclinical model as we have done in the current investigation.

A significant clinical obstacle in obtaining the cardiovascular benefits of statin therapy is SAMS. SAMS unfortunately impacts the physical and mental quality of life of those who experience these peripheral side-effects, and often ultimately results in non-compliance to statin therapy [47]. While discontinuance of statin therapy is indeed associated with reductions in SAMS, it is also associated with a concomitant rise in LDL-cholesterol, and invariably a rise in ASCVD risk [47]. While there is limited evidence on the molecular-level muscular outcomes of statin prescription in geriatric humans, the absence of changes in numerous functional and morphometric skeletal muscle outcomes observed within the current study is consistent with the work of Rengo et al. [48] who observed no evidence for statin-induced changes in muscle morphology at the molecular, cellular, or tissue level in men and women with a mean age of 70. In a cohort of octogenarian chronic statin users, there was also no evidence of greater phenotypic frailty despite significantly more comorbid conditions than matched non-statin users [49]. While detailed muscle analysis was not undertaken within this aforementioned study, and akin to what was observed by Rengo et al., evidence of preserved resilience and survival despite comorbid conditions in aged statin users is consistent with the observations present within the current investigation, namely, no between-group differences in: whole-muscle weight, individual muscle fiber size, presence of regenerating muscle fibers, and collagen within muscle sections. This collectively suggests absence of overt myopathic phenotype exacerbation at the cellular level with statin treatment.

The open field-testing procedures, implemented to evaluate general locomotor activity and willingness to explore, did not reveal a locomotive impairment with statin administration. These results are hypothesized to indicate the absence of myalgia (muscle pain) in the current model, which would have decreased ambulation and rearing, increased resting behaviours, and decreased total distance traveled during testing [50–53]. While there were significant pre-to-post study decreases in grip strength and ambulatory speed, no differences were present between treatment groups. Even when data points from RSV and ATV groups were combined and compared to CON animals, p = 0.49 for grip strength, p = 0.18 for average speed during ambulation, and p = 0.20 for fatigue, indicating the absence of a class-effect due to statins as a whole. The observed changes in muscle strength and travel speed are therefore likely a product of animal age; 26–28 months at study completion. As the upper limit of a rat’s life is approximately 36 months [16], it is hypothesized that one month of aging results in an exponential difference in overall health status during this phase of rodent life.

In humans, it has been reported that females are more sensitive to statin-induced myotoxicity and myopathy than males [54,55]. While reports in rodents mirror this myotoxic phenotype, cardiodynamics and open-field testing behaviours are affected similarly in male and female rodents following statin administration [56–58]. This broad spectrum of observations highlights the exclusive use of male rats within the current investigation as a limitation. An important consideration for future investigations should be the inclusion of both males and females within one’s study design.

Overall, by scrutinizing the cardiovascular system, intended to be protected from ASCVD risk with statin therapy, and the skeletal muscle, the tissue with the most clinically reported side effects of statin usage, this study allowed us to provide a comprehensive overview of the most clinically relevant effects of hydrophilic and lipophilic statins in a preclinical geriatric model necessitating statin therapy. In summary, while lipophilic ATV improved cardiodynamics and BP, hydrophilic RSV did not. Neither statin caused an overt myopathic or myalgic phenotype within the current investigation. While many preclinical investigations have reported the systemic and tissue-specific effects of statins in young rodent models, geriatric models, such as the one included herein, should be considered for more appropriate preclinical to clinical translation of the obtained results.
